# Awareness and Use of mHealth Apps: A Study from England

**DOI:** 10.3390/pharmacy5020033

**Published:** 2017-06-14

**Authors:** Reem Kayyali, Aliki Peletidi, Muhammad Ismail, Zahra Hashim, Pedro Bandeira, Jennifer Bonnah

**Affiliations:** School of Life Sciences, Pharmacy and Chemistry, Kingston University London, Kingston upon Thames, KT1 2EE UK; A.Peletidi@kingston.ac.uk (A.P.); muhammadirfan123@hotmail.com (M.I.); k0636978@kingston.ac.uk (Z.H.); pedrobandeira28@yahoo.co.uk (P.B.); jenny.bonnah@yahoo.com (J.B.)

**Keywords:** community pharmacist, mHealth, public health, public awareness of mHealth apps

## Abstract

**Purpose:** Mobile health (mHealth) solutions have become an inevitable element of the healthcare landscape. The recommendation and use of mHealth is important, but it is often underutilised. This study was conducted in England. It aimed to determine the use and recommendation of mHealth apps by pharmacists, the public’s perceptions of mHealth apps in general, and the awareness and use of health apps by diabetic patients in particular. **Methods:** The study used a mixed research approach, utilising a sequence of survey-based questionnaires with pharmacists and the general public, followed by semi-structured interviews with diabetic patients. **Results:** Pharmacists’ questionnaires revealed that 56% of the respondents were aware of health apps, 60% of which recommended them to patients. Over 76% of the individuals owned a smartphone. The types of applications that saw the most use from the general public were health and lifestyle apps (24%), social apps (19%), followed by news (18%). Although eight out of nine diabetic patients owned a smartphone, only three used diabetes apps. Diabetic patients also suggested an interest in using diabetes apps to aid in optimising care via the utilisation of visual aids, reminders, recording patient data, social coaching, and remote collaboration with healthcare professionals (HCPs), but time was seen as the biggest obstacle to using a diabetes mHealth application. **Conclusion:** Despite the growing number of mHealth apps, the level of awareness and usability of such apps by patients and pharmacists was still relatively low. Nevertheless, the majority who used health apps found them to be beneficial, and the public agreed that it helped them to live a healthier lifestyle. Therefore, health apps have great potential in health promotion. Pharmacists are ideally placed to promote them and make patients more aware of them. To increase the use of these apps, it is necessary to first increase awareness and knowledge of these apps, both to the public and to healthcare professionals.

## 1. Introduction

Mobile health (mHealth) is an essential element of electronic health (eHealth). MHealth is important because it makes healthcare practices accessible to the public through mobile communication technologies in a variety of ways (e.g., providing healthcare information, collecting health data, observing patients, etc.) [1,2]. MHealth apps also cover communication between users and healthcare systems (with call centers, and appointment and exact treatment reminders), monitoring and surveillance (with patient monitoring applications and surveys), and information access (with health records and medical diagnoses). In addition, mHealth aims to improve care by making health information easily accessible for patients with long-term conditions such as diabetes. The focus on mHealth is rapidly growing, due to the rise in production of smartphones and tablets that enable easier access to the Internet [3], making them an integral part of the healthcare landscape. There are more than 100,000 mobile applications today. The U.K. is becoming increasingly connected via the use of smartphones; research suggests that these are the most widespread of devices used by adults for online access. Since 2015, the number of adults who own a smartphone has increased to 71%. Among young adults under 34 years old, the percentage is even higher, estimated at around 90% [4].

The systematic review by Mossa et al. [5] recognised the importance of mHealth in medicine and healthcare. It was determined that mHealth could be used for disease self-management and remote monitoring of patients. For example, included in the review, a study by Marshall et al. [6] used a smartphone in order to enhance self-management of pulmonary rehabilitation in patients with chronic respiratory disease. A support system was designed to minimise the need for conventional labour-intensive services and to support enhanced management of patients’ rehabilitation programmes through improved self-involvement. The use of the platform allowed users to leave their home and to use a technology that they were familiar with. Furthermore, this provided important advantages over PC-based systems, which a lot of people did not have the necessary access to, nor knew how to use for their healthcare monitoring. The study concluded that this tool could be used in other patients who needed to exercise regularly, with the potential to increase self-management of their condition.

An international study conducted in four counties (U.S., Brazil, China and India) between March and June 2012, aimed to identify views of healthcare professionals, patients and customers regarding mHealth [7]. This end-user research was conducted in two different phases. The first phase included in-depth interviews in each of the participating countries on the views and current use of mHealth. The second phase of the research used online surveys to further explore topics discussed in the first phase of the research, including improvement of outcomes and quality of care, accessibility, affordability, and support of healthcare professionals in the use of mHealth. The participants of the study—89% of healthcare professionals (HCPs), 75% of patients, and 73% of customers—reported that mHealth solutions allowed patients and consumers to achieve health goals and improve health outcomes. The study demonstrated the perceived potential of mHealth to improve quality of care (50% for HCPs, 54% for patients, and 53% for consumers). Another important finding was that mHealth could support improved outcomes through behavioural changes, including compliance, with 53% of patients reporting that mHealth helped to increase their compliance to medications. In addition, 54% and 62% of patients and consumers respectively shared that mHealth could support diet and/or lifestyle changes. In addition, 61% of HCPs, 59% of patients, and 71% of consumers agreed that their usability of apps would increase if they were affordable. Finally, 52% of HCPs, 47% of patients, and 51% of consumers would download apps if they became simple to use. 

A systematic review by Dale et al. [8] found mHealth to be an important contributor to behaviour change intervention and disease management. The most investigated utilisation of mHealth has been the incorporation of text or short-message service (SMS), showing significant positive effects on health outcomes and/or behaviours. [8,9] Patients and consumers should be encouraged to talk to their HCPs about mHealth solutions, though these solutions could also benefit from being affordable, as well as simple, accessible and trusted. Community pharmacists are in a unique position to improve the patient care experience through mHealth, bridging the gap between patients and medicine. Through this, patients can remotely interact with pharmacists, thus reducing healthcare costs and promoting mHealth solutions. 

The majority of mHealth disease management interventions have focused on diabetes [8,9]. Patients living with diabetes experience difficulties associated with poor knowledge about their condition and the need to maintain a strict lifestyle. Diabetes, as a disease, is associated with many complications [10,11]; therefore, education, management, and control of diabetes is of vital importance [12]. MHealth could help diabetic patients make decisions necessary for optimal insulin dosing; maintain necessary lifestyle changes; and improve communication between patients, family members and allied healthcare professionals, such as pharmacists promoting self-management. Thus, this method could reduce hypoglycaemic episodes, with the potential to improve HbA1c (haemoglobin A1c) and the quality of patients’ lives. Moreover, self-management aims to involve patients in their long-term care, which empowers diabetic patients, improves self-efficacy, and minimises healthcare costs. Many studies have demonstrated the benefits of mHealth for both type 1 and type 2 diabetic patients. A meta-analysis on the effects of mobile phone interventions on glycaemic control [13] identified that strong evidence in regard to mobile intervention exists, resulting in statistically significant improvements in glycaemic control and self-management, particularly for type 2 diabetics. A reduction of HbA1c by a mean value of 0.5% (95% confidence interval) for both types was also observed. A difference between the two types was identified by a subgroup analysis, as 11 studies among T2DM patients reported a significantly greater reduction in HbA1c than studies among T1DM patients (0.8% vs. 0.3%; *p* = 0.02). 

The aim of this study was to explore the scale of awareness of mHealth apps and their recommended use by pharmacists for the public, as well as the public’s perceptions of mHealth apps in general. The study also explored the awareness and use of health apps by diabetic patients, the features desired for such apps by those patients, and how they perceived the role of the pharmacists and their care.

## 2. Methods

This study used a mixed research strategy involving two surveys in the form of questionnaires, one for the general public and one for community pharmacies, followed by semi-structured interviews with a specific patient group. Given that the number of people living with diabetes in the U.K. has tipped over the 4 million mark, patients with diabetes were targeted in order to grasp the scale of their awareness and usage of mHealth applications to manage their condition [12]. Ethical approval was acquired from Kingston University Ethics Committee. The study was conducted across London between 2012 and 2014. 

### 2.1. Community Pharmacy Questionnaire

The questionnaire was designed to identify and determine pharmacists’ views on the positive and negative aspects of promoting health via mobile health apps, as well as to understand their level of use in their daily practices. The questionnaire was piloted by 10 community pharmacists prior to distribution with no necessary changes. The questionnaire included open, closed, multiple-choice and Likert-scale-styled questions (Appendix A). The questionnaire was divided into the following sections: provided services, awareness and recommendation of health apps, and awareness and usefulness of the Centre for Pharmacy Postgraduate Education (CPPE) guide titled the “Apps for Pharmacy” [14]. All selected pharmacies were provided with questionnaires, either distributed by hand or by mail with self-addressed envelopes. A certain timeframe to complete the survey was given, and pharmacists were reminded of approaching deadlines via phone call.

### 2.2. Sampling

Convenience sampling was used due to proximities. The following London Boroughs were included in the study: Kingston, Sutton, Greenwich, Hackney & City, and Tower Hamlets. The number of community pharmacies in London, according to the Tower Hamlet Pharmaceutical Need Assessment (PNA) report 2015, was 1846 [15]. The combined number of community pharmacies in the London boroughs included in the study was 288 (32 in Kingston, 82 in Sutton and Merton, 61 in Greenwich, 65 in Hackney & City, and 48 in Tower Hamlets). The sample size was calculated using the Raosoft sample size calculator [16]. The total sample size calculated for the Boroughs included in the study was 165 at a 95% confidence level and 5% margin of error. To achieve the sample size, 40 pharmacies in each of the selected areas were approached.

### 2.3. Public Questionnaire 

The questionnaire for the public, consisting of open, closed and Likert-scale-styled questions, determined whether participants owned a smartphone, used apps and were aware of mobile health apps, and whether they had any specific medical conditions. Basic demographic-related questions were also included (Appendix A).

### 2.4. Sampling 

The questionnaire was piloted prior to distribution on a sample of 20 members of the public, whereupon it was found to be viable. A convenience sample was used to choose the locations of the survey. Locations included three London Boroughs: Kingston, Tower Hamlets, and Merton. While volunteers in Kingston and Tower Hamlets were approached on high streets, volunteers in Merton were approached at coffee shops in Raynes Park, the Raynes Park Health Centre, and Centre Court, Wimbledon. The sample size was calculated based on the total population of the selected boroughs (671,900), again using the Raosoft sample size calculator [16]. The calculated sample required was 380 at a 95% confidence level and 5% margin of error. Thus, 380 volunteers, aged 18 and above, were randomly approached to participate, but only 154 finally agreed to complete the questionnaire.

### 2.5. Public Interviews 

A qualitative approach of semi-structured, one-on-one interviews was used to determine diabetic patients’ perceptions and awareness of the use of mobile applications for diabetes. Volunteers were recruited by contacting diabetic charities, such as Diabetes UK, and local patient groups. The semi-structured interview schedule consisted of four sections on diabetes: the disease in general, management challenges, education, and smartphone usage (Appendix A). The interview schedule was piloted with one diabetic patient, resulting in no changes. Participant information sheets were distributed describing the purpose of the study. Interested participants were given consent forms, which were returned, signed, before the interviews. Nine participants in total (four type 1 and five type 2 diabetic patients) were recruited. Recruitment was continued until data saturation was reached. Data saturation occurred early at the sixth interview. However, all interviews were included in the analysis. The interviews took place across London in specific locations mutually agreed upon by researchers and participants. Each interview lasted approximately 60 min. All interviews were audio-recorded and then transcribed verbatim. Before analysis, transcribed data was anonymised and participants were given pseudonyms. Pseudonyms were devised, for example, by combining the type of diabetes with the patients’ codes (e.g., “T1DM1”), and were applied to all participants. Thematic analysis was conducted to identify themes. The transcripts were coded independently by two researchers using NVivo qualitative data analysis Software; QSR International Pty Ltd. Version 11, 2015. The codes and themes generated were then checked and agreed upon by the research team. 

## 3. Results

### 3.1. Community Pharmacy Questionnaire

In total, 200 community pharmacists, 40 in each area, were approached to participate in the questionnaire; however, 95 pharmacists responded in total. More specifically, the most responses were obtained in Kingston (39%; *n* = 37), followed by Tower Hamlets (22%; *n* = 21), Greenwich (19%; *n* = 18), Sutton (11%; *n* = 11), and finally Hackney & City with the least number of responses (9%; *n* = 9).

Concerning whether pharmacists were aware that mHealth apps were available for smartphones, the response was nearly equal between those who were aware (56%; *n* = 53) and those who were not aware. Of those who were aware of these apps (*n* = 53), 60% of them (*n* = 32) recommended them to the general public.

The questionnaire also delved into questions regarding the CPPE guide. In terms of awareness, the majority, 72% (*n* = 68), reported that they were not aware of the CPPE guide. Of those pharmacists who were aware of the guide, 67% had read it (*n* = 18), and 33% had not (*n* = 9). Once again, of those pharmacists who knew of the guide, 63% (*n* = 17) found the guide useful, whereas 37% (*n* = 10) did not. Finally, of those who were aware of the CPPE guide and had read it, who recommended downloading and using apps, 78% (*n* = 14) had downloaded and used them, whereas 22% (*n* = 4) had not.

### 3.2. Public Questionnaire

Overall, 154 members of the public responded. Demographic information for the participants is outlined in Table 1 below. Age distribution was equal in the sample, but in terms of gender, females responded more than males.

Overall, the majority of respondents owned a smartphone (76%; *n* = 117) compared with those who did not (24%; *n* = 37). Those who owned a smartphone downloaded a variety of available mobile applications. Most of the respondents downloaded health and lifestyle apps (24%) and social apps (19%), followed by news apps (18%) and games (17%). Other downloaded apps included books (11%), educational apps (11%), business apps (8%), and some apps (2%) that did not belong in any other category.

Respondents who downloaded health and lifestyle apps were asked to provide more details; only 21 out of 29 respondents did. Among the apps downloaded were fitness apps (*n* = 7), and diet and nutrition apps (*n* = 6). Users of such apps found them to be beneficial and helpful for maintaining a healthier lifestyle. A good number of participants downloaded BMI and alcohol units apps (*n* = 4), while others downloaded smoking cessation apps (*n* = 3), and one participant downloaded apps related to vascular risk assessment.

Finally, participants who owned a smartphone (76%; *n* = 117) were questioned on whether they would use a health app that was not backed by a recognised health authority such as the NHS. Most of the respondents (42%; *n* = 49) disagreed with the statement “I would use a health application that is not backed up by a recognised health authority such as the NHS”, some (32%; *n* = 38) agreed or strongly agreed, and a number of them (26%; *n* = 30) were neutral.

### 3.3. Public Interviews

Nine participants were interviewed in total: four type 1 diabetic patients (T1DM) and five type 2 diabetic patients (T2DM). Each participant was assigned a consecutive code. The T1DM patients were coded “T1DM1” to “T1DM4” and the T2DM patients were coded “T2DM1” to “T2DM5”. 

Four themes were identified: pharmacist’s role in diabetic care, use and features of diabetes apps, barriers for use of apps, and the value of healthcare professionals.

### 3.4. Pharmacist’s Role in Diabetic Care

Participants felt that pharmacists play a major role in their healthcare. All those who were interviewed (*n* = 9) acknowledged that pharmacists were necessary for dispensing medication and providing them with advice. In addition, eight participants had a positive view of their pharmacist because they had known them for a long period of time. One participant (T2DM3) reported: “*Erm, my pharmacist, he is actually a really nice guy, every time I go he does ask me how I’m doing, and that’s probably because I’ve been going to him for the last twenty years. So that’s why I think he’s concerned about me, he’s seen me from a young woman, and so yes, and that’s only because I know him very well*.”

In contrast, one participant (T1DM3) outlined that they felt that their pharmacist’s only purpose was to dispense their medication. In addition, they theorised that pharmacists could play a bigger role in type 2 diabetic patients’ care as opposed to in type 1 diabetic patients’ care: “*Their role is really just to dispense. Apart from that, I don’t think they have a very interactive role maybe because I don’t interact with them. I-I don’t think they can for type 1, maybe for type 2 it is a bit different.*” Conversely, another participant (T2DM1) mentioned that their pharmacist cared about their condition, and that pharmacists could advise them based on their condition in order to minimise complications: “*Yeah, my local pharmacist is brilliant because he knows I’m diabetic. Like two or three days ago I went to get cough syrup, and he knows I can’t have normal cough syrup and I can’t have these things with sugar, so he’s very good, he said ‘No, you can’t have this one,’ so he’s very good like that.*” In addition, aside from dispensing medicines and offering general advice, some participants did in fact mention that their pharmacist was concerned about their well-being and that pharmacists informed participants when they were available if they needed advice. Many participants mentioned how pharmacists were “*the first port of call*” and could be contacted without an appointment, unlike a GP.

When delving further into the discussion as to whether pharmacists had any other roles, none of the participants mentioned any intervention steps such as a medication review or in-depth counselling, but one T2DM patient mentioned that the pharmacist did conduct an “*interview*” with them and that the pharmacist encouraged them to come back every year. 

### 3.5. Use and Features of Diabetes Apps

All participants, both type 1 and type 2 diabetic patients, who owned a smartphone (*n* = 8) stated that they mainly used these devices for messaging and/or social media. Participants reported: “*I usually use WhatsApp and the calendar*,” (T1DM2); “*Oh, I use WhatsApp, Facebook, that’s it […]*” (T2DM1); “...*what do I use the most? I think WhatsApp at the minute...*” (T2DM2); and “...*erm, checking my emails, erm, […] and text messaging..*.” (T2DM3). 

Only three participants stated that they used apps for diabetes; the two mentioned were the “Diabetes UK” app and “Carbs and Cals”. From their answers, it can be determined that such applications could be very beneficial to them, and could help them to manage their condition. One participant reported “*For a period I used that Diabetes UK app that monitors your blood sugars and plots a graph...*” (T1DM1). In addition, another mentioned “*There’s the Diabetes UK one which I found very useful in that one you can monitor, you can put your units in, your glucose, you know, review so when you go to the hospital, […] I’m not going to remember twice a day when to inject, or twice a day when to take tablets or whenever I do my glucose monitoring [so it is beneficial to use such an app]*” (T2DM2). 

However, participants who had tried diabetes applications revealed that they stopped using them, stating: “I found it to be, like, pointless, to be honest” (T1DM1); “Carbs and Cals, yeah. That was the application. So yeah, maybe because I didn’t remember it, I didn’t use it that much […]” (T1DM3).

In terms of what participants felt would be useful to incorporate into a diabetes application to aid condition management, most of them (*n* = 6) agreed that there was a need for reminders, as these would be very useful for managing their diabetes. For instance, T1DM2 outlined that reminders for “*blood glucose*” monitoring and “*reminders for the night time insulin*” would be helpful, as well as “*reminders for appointments*”*.* Such a response was reciprocated by T1DM3, who felt that “*something to remind you to keep checking, to keep your diet on strict regime*” was useful, and also by T2DM3 who said, “*reminders you know to take your insulin, or test your sugar*”, *admitting that* “*that’s the biggest hurdle*”*.*

Moreover, several participants also responded that they would like to use visual aids for education and monitoring in an mHealth application: “*[Videos], it would definitely help*” (T1DM2); “*...if you had the graphs, then you could base your interpretation on the graphs. You can see if there’s a wide variation or if there’s a constant range*” (T1DM3); and “*So yes something on a graph will show you progress*” (T2DM3).

Two participants also detailed that the use of a function that helped them with identifying the effect of food on their blood glucose would be of benefit. T1DM3 stated, “*If you’re stuck between two foods to eat, telling you that this is six units or bad for your glucose levels…. or this one can enter the two and help you a little bit*”; and T2DM2 felt that a quiz to “*test your knowledge about foods and how they affect your glucose levels*” would be beneficial.

Moreover, the majority (*n* = 7) of the interviewees encouraged the use of social media to share diabetes-related experiences in a diabetes mobile application, and eight felt that a chat room or a forum for social interaction within the app would be beneficial. Such a proposal could potentially help diabetic patients support each other and exchange different perspectives on their conditions. For example, participant T1DM2 felt that having “*more people within these chat rooms*” would help provide advice in order to “*help each other*”*,* and the participant also detailed that “experience is definitely a big thing.” Furthermore, another participant (T2DM1) felt that such a function would be useful to “*discuss what is best thing to eat or can you find alternatives*”*.*

### 3.6. Barriers for Use of Apps 

Participants were questioned regarding what barriers they faced, as well as the reasons they did not use these apps regularly. Barriers such as lack of awareness, time constraints, finding such apps pointless or an inconvenience, limited available functions, and plain forgetfulness on their parts were amongst the reasons that such apps were not used frequently.

A lack of awareness was a big barrier stated by the interviewees. Most participants were not aware about the existence of diabetes applications. Interviewees reported the following: “*Are there any?*” (T1DM2); “*Erm, I’ve never really experienced, you know, an app like that before, but I think if there was an availability of one, I think I would definitely implement it into my life...*” (T1DM4). 

By evaluating participants who considered using a mHealth application (*n* = 7), five of these individuals stated that time was one of the biggest barriers to using a mHealth application on a regular basis: “*Time management. Depends on the person, if he’s willing to put the time in*” (T1DM3); “*[There are] more priorities than using the smartphone for managing diabetes*” (T1DM4); “*Then you have to enter [blood glucose results] on your app, why waste my time on that entry, I don’t see the point of that*” (T2DM1); and “*Just time*” (T2DM2). Participant T1DM2 acknowledged that initially time was a barrier, but that “*[It] would be something I would have to get used to. It would just be normal, just like diabetes as a whole*”. Therefore, despite time being an initial limitation, some of the participants stated that once they had become more proficient with an application, they could better monitor their condition. 

In addition, another one of the limitations outlined for such a function was privacy and security issues, as mentioned by participant T1DM4, who said that “*people’s privacy should be respected*”.

### 3.7. The Value of Healthcare Professionals

All participants (*n* = 9), regardless of whether they used diabetic apps or not, encouraged sending their mobile phone data to HCPs. Many individuals responded with the benefits of such a function, reinforcing that it would save time, allow for quicker responses, and even help HCPs themselves. One response outlined: “*It would be helpful if there was universal software for all the patients*” (T1DM1). Other respondents saw the benefit of having electronic access by HCPs, stating: “*It would probably help the professionals more*” (T1DM2); “*allowing them greater detail will in actual fact aid that process of treating you*” (T1DM3); and “*the reason that we are collecting all this data is to help the healthcare professional*” (T1DM4).

One participant (T2DM1) stated that such a function would save healthcare professionals’ time (“*...because sometimes they’re busy...*”) and provide them with effective advice via “a phone consultation” without needing to book an appointment. The participant concluded, “*It would be a brilliant way of dealing with day to day busy people*.”

Another participant (T2DM3) saw the practical benefit of such a function compared to the traditional methods of recording blood glucose. They initially stated: “*Never, never did it [record their blood glucose results] and then I would quickly get my, erm, blood sugar monitor and quickly write it in.*” The participant also outlined the challenge of logging blood glucose results, as they would have to “*go back, back, back and try and remember times and things*”. The participant concluded that such a function “*would be great because you take it with you they can have a look*”*.*

## 4. Discussion

This study focused on how aware pharmacists were of mHealth apps and whether they recommended their use, as well as how the general public perceived such apps. For this purpose, two questionnaires were distributed, one to community pharmacists and one to the general public, followed by semi-structured interviews with diabetic patients. Despite the wide range of different services that pharmacists provided, they did not take advantage of the available mHealth apps as an additional tool to support these services. The results have indicated that almost half of the participating pharmacists were not aware of mHealth apps for health promotion and needed to be educated in this area. These results confirm other research findings, which showed that the majority of health apps available on smartphones are underutilised [17]. The CPPE guide launched in 2011 for available applications highlighted 40 health apps that pharmacists could use and/or recommend to their patients [14]. An important outcome was that most of the participating pharmacists who responded to the questionnaires were not aware of and had not read the CPPE guide on health apps. Of those who were aware of and had read the guide, the majority of them found the guide to be useful and indicated that they would download and use health apps recommended by the guide. The results showed that pharmacists need to be properly educated to enhance their awareness of the guide and the health apps recommended by it. Increase of awareness and further education may help pharmacists to use and recommend more mHealth apps in their everyday practices. The CPPE, which is responsible for the continuous professional development of pharmacists, needs to focus on engaging pharmacists so that they can become familiar and comfortable with emerging technology in their everyday practices for self-development and health promotion. 

Most members of the general public that answered the questionnaire owned a smartphone. This finding was reflective of the diabetic patients interviewed, with most owning a smartphone. Members of the public were asked about what kind of apps they downloaded on their smartphones most frequently. The majority of them were social apps, such as Facebook and Twitter, and only a small number of respondents had downloaded health apps. Of those who downloaded health apps, most of the apps were associated with fitness, nutrition and diet; the same was determined by the Krebs et al. study [17], which identified the usage of health apps between smartphone owners. Fitness and nutrition were the most common types of health apps used, with most respondents using them daily. Counselling and health information to promote lifestyle changes could also be incorporated into games and education apps to increase the use of such technologies. In addition, companies could provide an innovative and interactive way of designing new apps to satisfy their users. This connection represents an encouraging field for the market of app development. 

Moreover, interviewees had conflicting views on pharmacists and their roles in helping them to manage their condition. This varied between type 1 and type 2 diabetic patients. They all felt that pharmacists played a crucial role in their healthcare, as all acknowledged that pharmacists were vital in dispensing medication and providing advice on its usage. However, the interviewees based their responses on their personal long-term relationships with their pharmacists, and not because they believed that pharmacists played a more advanced role. Despite pharmacists being recognised for their importance in diabetes care, only one participant mentioned that pharmacists did indeed play a clinical role in diabetes care.

Despite the effects of mobile phone interventions on glycaemic control that previous studies identified [13,18], resulting in statistically significant improvements in its control and self-management, this study determined through interviews with diabetic patients that very few of them were aware of health apps, apart from 3 of the participants who owned a smartphone, the remainder admitted that they did not even know such apps existed.

Unfortunately, of the participants who knew of and had tried diabetes mobile apps, none used them daily. The main shortcoming mentioned by participants was the fact that, although having a diary in which to record blood glucose levels was practical, inputting the data was tedious and time-consuming. Therefore, the main barriers in the use of diabetes apps were time constraints and usability. Firstly, it took time to learn how to use the application; secondly, it took too long to input data and it was also inconvenient to maintain and update the app daily with a busy schedule. Studies also confirmed that some common reasons people did not keep downloaded apps were because of costs, a lack of interest, and a concern about apps collecting their data. Individuals who commonly used health apps tended to be younger and have higher incomes, and were more educated [17,18]. 

Participants expressed their desire for an app that had features that included visual functions. Trends through graphs, videos, games, quizzes and comparative functions identifying the effect of food on glycaemic control were among the desired features of a mHealth app. Most studies agree with these positive findings, identifying the impact of visualisation and interactivity in diabetes education [19]. However, another study identified that, although such functions may help in understanding diabetes, they are limited in clinical benefits (HbA1c improvement; *p* = 0.38) and improvements in theoretical knowledge (*p* = 0.82) [20]. 

In terms of the participants’ perceptions towards mHealth and diabetes, social networking was another aspect that was encountered as it could improve self-efficacy and self-empowerment [21,22]. Using social media was desirable for participants in order for them to share their diabetes-related experiences. As mentioned in the Labate study [23], people with diabetes are given entirely new avenues to explore through online communities. Through communicating with others who experience similar situations, and sharing life experiences with each other, they can become encouraged to be more open about their struggles and change their habits. In fact, this can also promote communication with their healthcare professionals, thus enabling diabetic patients to take an active role in their treatment and make more informed decisions. This gives them more self-confidence, which in turn means that they can apply their newly-acquired knowledge to help properly manage and control their diabetes. Another study by Moskowitz et al. [24] indicated that peer health coaching had a larger effect on lowering HbA1c in patients with low levels of medication adherence and self-management support than in patients with higher levels. Peer health coaching interventions may be most effective if those who are targeted are high-risk diabetic patients with poor glycemic control and poor self-management and medication adherence.

Medication and appointment reminders were also reported to be desired functions of mHealth apps. The Krishna et al. study [25] reported that education and management of diabetes through text and telephone reminders have been shown to enrich knowledge, increase the frequency of self-care behaviours in diabetic patients, and improve health outcomes for patients who needed regular care and monitoring, as well as self-care management. Because monitoring and support from a healthcare provider play important roles in achieving patients’ desired clinical goals, the use of mobile phones, especially through text messaging, is a step forward in achieving the health and quality of life for those with diabetes. The global nature of mobile phones provides mobility and flexibility so that care can be provided wherever the patient is [25]. In addition, a Cochrane review [26], which explored the effectiveness of mobile phone text message reminders, concluded that such reminders improved the rate of attendance at healthcare appointments, as opposed to having no reminders. While text reminders were as successful as phone call reminders, they were significantly more cost-effective.

The study also discovered that participants were open to data sharing options with a HCP through mHealth apps; they realised the advantage of having a data sharing option for both healthcare professionals and patients, primarily in the context of these interventions being inclusive of clinical improvements, positive behavioural improvements, and increasing effective treatment levels while reducing care costs. It must be noted, however, that sharing data has limitations. It is ineffective if the participant is unwilling to be consistent and honest, and it also loses its effectiveness if it is overused [27,28].

In conclusion, this study identified a lack of awareness and use of health apps by the public—including those with a long-term condition—despite their benefits. This is exacerbated by HCPs lacking sufficient knowledge and failing to recommend such apps to their clients and patients, despite the benefits. The limitation of this study lies in the fact that it reflects the views of pharmacists and members of the public between 2012 and 2014, and so the uptake, awareness, and range of apps available may have increased since then. Other limitations included the low response rate for both pharmacists and the public questionnaire, as well as in the interviews conducted, thus limiting the generalisability of the findings. Another limitation is the possibility of selection bias. The characteristics of participants who agreed or not were not explored in the study. 

Nevertheless, this study highlights the acceptance of health apps by the public. In addition, it outlines the desired features of such apps so that their use and uptake can benefit patients, particularly those who are diabetic.

## Figures and Tables

**Table 1:** Personal Demographic Information.

**Gender Distribution (*n* = 154)**				
Sex	Frequency (n)		Percentage (%)	
Males	73		47	
Females	81		53	
**Age Distribution (*n* = 154)**				
Age range	Frequency (n)		Percentage (%)	
<40 years	77		50	
>40 years	77		50	
**Medical Conditions (*n* = 39/154)**				
	Hypertension	Dyslipidaemia		Diabetes
Frequency (n)	16	14		9
Percentage (%)	41	36		23

## Appendix A

1. Pharmacist’s Questionnaire
              **Questionnaire for pharmacist**
  1. Are you aware of the availability of health apps (applications) for smartphones?
    - Yes
    - No
  2. Do you recommend health apps (applications) for smartphones to the public?
    Yes      No    
  3. Are you aware of the CPPE (Centre for Pharmacy Postgraduate Education) guide to health apps for pharmacists?
    - Yes
    - No
  4. Do you read the CPEE guide?
    - Yes
    - No
  5. How do you rate the usefulness of the guide for your practice?
    - Very useful
    - Useful
    - Somehow useful
    - Unuseful
2. Public’s Questionnaire
  1. Do you own a smartphone?
    ☐ Yes
    ☐ No
  2. Are you aware of the availability of health apps (applications) for smartphones?
    ☐ Yes
    ☐ No
  3. What type of applications do you download?
    ☐ Games
    ☐ Education
    ☐ Books
    ☐ News
    ☐ Social
    ☐ Health and Lifestyle
    ☐ Business
    ☐ Other
  4. If you download a health app, what type of such app do you download?
    ☐ BMI/alcohol unit
    ☐ Smoking cessation
    ☐ Vascular risk assessment
    ☐ Fitness
    ☐ Diet and nutrition
  5. Please tick the box that best describes what you think.
    “I would use a health application that is not backed by a recognised health authority such as the NHS.”
    ☐ Strongly Agree
    ☐ Agree
    ☐ Neutral
    ☐ Disagree
    ☐ Strongly Disagree
    Demographics
  6. Do you have any of the following medical conditions? (Tick as many as appropriate)
    ☐ Diabetes
    ☐ High blood pressure
    ☐ High cholesterol
    ☐ None of the above
  7. What is your gender?
    ☐ Male
    ☐ Female
  8. What age group are you?
    ☐ <40 years old
    ☐ >40 years old
3. Diabetic Patients’ Interview Schedule

Welcome and thank you for taking part.

Introduce interviewer.

The purpose of this interview is to ask you about your perceptions on diabetes mobile health applications designed to improving the knowledge and management of diabetes. The interview will last for approximately 60 minutes. You can take a break at any time during the interview. Be assured that your responses will be anonymous and will in no way be linked to you or your identity. Please do provide me with your opinions as best you can and understand that there is no right or wrong answer. The interview will be recorded and the recordings will be deleted once the interview is transcribed, are you ok with this?

Can I please ask you to read the consent form detailing the research involved and ask you to sign it, to confirm that you understand the study and are willing to take part?

Thank you.

Do you have any questions before we begin?

1) **What type of diabetes do you have and how long ago were you diagnosed?**
2) **Who is currently involved in your diabetes care?**
  Prompt if required: In this question, I am referring to those that have a direct influence on your diabetes care such as diabetic nurses, doctors, family members and other individuals such as friends.
3) **Do you feel that pharmacists play a role in your healthcare?**
4) **What role does a pharmacist play?**
  Prompt if required: Dispensing of medicines, providing relevant advice, provision of essential services, signposting to appropriate healthcare professionals. etc.
5) **How familiar are you with smartphones?**
6) **Do you currently own a smartphone?**
7) **What features of the smartphone do you use daily?**
  Prompt if necessary: Calendar, internet, social networking, e-mail, games, applications.
8) **What types of applications do you use the most?**
  Prompt if necessary: Social network applications, office applications, healthcare applications, music applications, applications to help organise better, etc.
9) **Of all the time spent using your smartphone, what features would you say takes up the most time?**
  Prompt if required: Do you spend most of your time web browsing, messaging, on social media, playing games, etc.
10) **What is your opinion on the use of diabetes mobile health applications to better manage diabetes?**
  Prompt if required: These are a form of mobile applications specially tailored for diabetes care. They contain features such as a blood glucose meter log, medication and diet logs, weight management and exportation of data.
11) **Have you used any form of electronic management with regards to your diabetes?**
  Prompt if required: Use of electronic diaries, contacting your diabetic nurse or consultant via e-mail or phone, etc.
12) **Have you used a mobile smartphone application to better manage your condition?**
  Prompt if required: Examples of such include the Diabetes Tracker application, Carbs and Cals application, IBG iPhone application, etc.
13) **What features of the application/s did you feel had not aided you in better managing your condition?**
14) **Do you feel that there are any barriers to you using a mobile health application?**
  Prompt if required: Barriers such as time, convenience, reluctance to change management habits, user friendliness of the application, whether it is too complex to use, etc.
  If no, go to next section.
15) **With regards to diabetes management, what features do you think will be most useful in a diabetes mobile health application?**
  Prompt if required: Features such as diary of blood glucose levels, diary of food intake, inputting events such as exercise, graphs of blood glucose levels, reminders for appointments, checking blood glucose, taking medication, etc.
16) **How do you feel about the access of healthcare professionals to your data that is logged onto a mobile health application in order to provide extra support or healthcare advice?**
  Prompt if required: Data such as blood glucose results, changes in weight, doses of medication taken, etc.
17) **What is your opinion on the use of social media to share diabetes related experiences with others?**
  Prompt if required: Such examples of experiences that you feel that only diabetics can understand or experiences which may aid others in living with diabetes.
18) **Further to this, what is your opinion about the addition of a chat room or forum to aid social interaction?**
19) **Finally, what do you think about motivational messages to help you to achieve diabetes related goals?**
  Prompt if required: For example, images or messages of congratulations if you reach your target blood glucose, target weight, target HbA1c, meet the calorie target of your diet, take your medication on time, etc.
